# Impact of Greater Surgeon–Anaesthetist Familiarity on Post-Operative Outcomes in Upper Gastrointestinal Cancer Surgery: A 10-Year UK Tertiary Cancer Centre Experience

**DOI:** 10.3390/cancers18162621

**Published:** 2026-08-14

**Authors:** Nikhil Manish Patel, Kai Tai Derek Yeung, Pranav Harshad Patel, Joseph Doyle, Torsten Beutlhauser, John Williams, Michelle O’Mahony, John Schutzer-Weissmann, Ravishankar Raobaikady, Matthew Hacking, Richard Gordon-Williams, William Allum, Mohammed Asif Chaudry, Ricky Harminder Bhogal, Sophie Uren, Sacheen Kumar

**Affiliations:** 1 Department of Upper Gastrointestinal Surgery, The Royal Marsden NHS Foundation Trust, London SW3 6JJ, UK; 2 The Upper GI Surgical Oncology Research Group, Division of Radiotherapy and Imaging, The Institute of Cancer Research, London SW7 3RP, UK; 3 Department of Anaesthesia and Perioperative Medicine, The Royal Marsden NHS Foundation Trust, London SW3 6JJ, UK; 4 Department of Upper Gastrointestinal Surgery, Digestive Disease and Surgery Institute, Cleveland Clinic London Hospital, London SW1X 7HY, UK

**Keywords:** dyad, post-operative outcomes, teamwork, upper gastrointestinal surgical oncology

## Abstract

**Background**: Greater familiarity between surgeons and anaesthetists is associated with better teamwork and patient safety. We investigated whether the volume of major UGI cancer resections the same surgeon and anaesthetist perform together influences post-operative outcomes at our tertiary specialist cancer centre. **Methods**: A 10-year retrospective cohort study was conducted via propensity score matching analysis (PSM). Consecutive adults undergoing elective surgical resection for primary UGI carcinoma and colorectal liver metastases from January 2014 to December 2024 were included. Cases were performed by surgeon–anaesthetist dyads composed of five Consultant Surgeons and 34 Consultant Anaesthetists. The primary Consultant Surgeon and Consultant Anaesthetist, the Charlson co-morbidity (CCM) score, and Clavien–Dindo (CD) complications were recorded. Cases were matched for age and CCM score. **Results**: A total of *n* = 792 cases were included, which became *n* = 546 after PSM. The median number of cases performed per dyad was 23, and the median CCM score = 5. Dyads were stratified into high (≥23 cases/dyad) and low volume (<23 cases/dyad), and cases into high (CCM ≥ 5) and low risk (CCM < 5). High-volume dyads had a lower incidence of post-operative complications when adjusted for PSM (35.2%) compared to low-volume dyads (42.8%) (*p* = 0.829). Probability of CD grade III complications among low-volume dyads increased with a rise in CCM (*p* = 0.224), but not in high-volume dyads. **Conclusions**: Greater familiarity between surgeons and anaesthetists may develop by operating on more cases together. This study has highlighted a trend suggesting that high-volume dyads could safely perform UGI resections on higher-risk cases without significant increases in post-operative complications. Dyad volume should be considered in operative scheduling for UGI cancer surgery.

## Simple Summary

The working relationship between the surgeon and the anaesthetist is important in ensuring good post-operative outcomes. Greater familiarity between surgeons and anaesthetists may be achieved through performing more cases together. We carried out a 10-year study at our specialist cancer centre to investigate whether the more major upper gastrointestinal (UGI) cancer resections the same surgeon and anaesthetist perform together, the better the post-operative outcomes. We demonstrated an association between surgeon–anaesthetist dyad volume and post-operative outcomes. We observed a trend suggesting that the same surgeon and anaesthetist pairing (dyad) could perform a higher number of UGI cancer resections in higher-risk patients without a significant increase in inpatient mortality or length of stay. We therefore recommend that surgeon–anaesthetist dyads should be considered in pre-operative planning for major UGI cancer surgery, especially in cases of higher-risk patients.

## 1. Introduction

A close working relationship between a surgeon and an anaesthetist, termed a ‘dyad,’ has been reported to be mutually beneficial for patient safety and teamworking [1]. Recent evidence indicates that consistently operating within specific surgeon–anaesthetist dyads may have a significant influence on post-operative outcomes [2]. This is of particular importance in patients undergoing complex surgery for upper gastrointestinal (UGI) tract cancers. Previous research has identified a strong correlation between the volume of UGI tract procedures performed and post-operative outcomes [2]. The repetition of a standardised approach to major UGI cancer resections, including oesophagectomy, has been shown to improve clinical and oncological outcomes [3]. However, patients undergoing complex UGI surgery are also more likely to have better outcomes when operated on by surgeons routinely performing these procedures in high-volume centres [4]. This ‘surgeon volume–outcome relationship’ has been extensively studied and reported in numerous countries including the United Kingdom (UK), the United States, and Germany [4,5,6,7]. The index of post-operative outcomes universally measured includes the length of stay (LoS), incidence of post-operative complications according to the Clavien–Dindo (CD) classification, rate of readmission to hospital, and 30- and 90-day mortality [8,9].

Surgical resections for UGI tract malignancies are complex procedures associated with increased peri-operative risk [10]. Overall incidence of post-operative morbidity can be as high as 59% following oesophagectomy and 40–50% after pancreaticoduodenectomy [11,12]. Following the observed volume–outcome relationship, the centralisation of cancer services in the UK has resulted in the redistribution of these cases to high-volume centres, which have the appropriate or specialised peri-operative medicine and anaesthetic and critical care support [13]. This may provide patients with speciality specific pre-, peri-, and post-operative care from experienced healthcare teams, minimising surgical morbidity and improving outcomes, including overall survival [14,15].

The impact of anaesthetists with experience in peri- and intra-operative management of patients with resectable UGI and hepato–pancreato–biliary (HPB) cancer has been studied in comparatively less detail than with surgeons [16,17]. In a national cohort study of 8096 adults who underwent oesophagectomy, pancreatectomy, or hepatectomy for cancer, Hallet et al. demonstrated that patients who received intra-operative care from high-volume anaesthetists, defined by anaesthetising for at least six of these three types of procedure per year (75th percentile), had a lower risk of 90-day major morbidity compared to low-volume anaesthetists, who were those performing fewer than six such procedures annually (25th percentile) [18].

Teamwork is at the crux of providing good patient care, and the operating theatre is an example where positive team dynamics and clear communication between the surgeon, anaesthetist, and theatre staff are of paramount importance [19]. Close collaboration in the peri-operative management of patients has been suggested to improve patient safety and post-operative outcomes [20,21]. Within the context of complex UGI and HPB cancer surgery, strict peri-operative anaesthetic management of fluids, pharmacological cardiovascular control, and transfusion of blood products are examples where clinicians within an experienced dyad may influence a patient’s clinical outcome from surgery. This is typically achieved through common teamworking on a high volume of complex UGI and HPB cases, with the repetition of critical steps building familiarity and incrementally improving patient outcomes [1].

Having read national-scale studies by Hallet et al., we aim to investigate if similar associations between surgeon–anaesthetist dyad volumes and post-operative outcomes in major UGI and HPB cancer surgeries exist at a local level at our tertiary specialist cancer centre in the UK [9,18]. We hypothesise that the greater the number of cases a surgeon–anaesthetist dyad perform together, the lower the risk of adverse post-operative outcomes among patients undergoing elective resectional surgery for UGI and HPB cancers within our foregut unit.

## 2. Materials and Methods

### 2.1. Patient Population

A retrospective cohort study of consecutive adult patients (>16 years old) that underwent elective surgical resection for upper gastrointestinal (UGI) tract malignancy at our tertiary specialist cancer centre over a ten-year period, from January 2014 to December 2024, was performed.

All consecutive adult patients that underwent major oesophago–gastric (OG) or hepato–pancreato–biliary (HPB) resection for adenocarcinoma, squamous cell carcinoma, or colorectal liver metastases (CRLMs) within the specified period were included. The majority of procedures were performed with curative intent. Resections performed for any other malignancies, including gastrointestinal stromal tumours and neuroendocrine tumours, were excluded. All cases were discussed in a joint UGI multi-disciplinary team (tumour board) meeting. All surgical procedures were performed at our cancer centre, and all patients were admitted routinely to the critical care unit (CCU) post-operatively. Post-operative management plans for each procedure included in this study are broadly standardised. However, any changes to components of post-operative care were specific to each case and were made at the discretion of the Consultant Surgeon. Consultant Anaesthetists underwent specialist fellowship training prior to commencing independent practice at our centre. The National Health Service Health Research Authority online decision tool was used, which confirmed that ethical approval was not required to carry out this study.

Primary outcomes were post-operative lengths of stay (LoS) in the CCU, total inpatient LoS, and complications according to the Clavien–Dindo (CD) classification. Secondary outcomes included 30- and 90-day mortality. The study has been reported in accordance with the ‘Strengthening the Reporting of Observational Studies in Epidemiology’ (STROBE) statement guidelines [22].

### 2.2. Clinical Data

Data were extracted from a prospectively maintained database of all patients undergoing surgical resection for UGI cancers and CRLMs and electronic patient records. The primary operating Consultant Surgeon and Consultant Anaesthetist were recorded in all cases. Pre-operative clinical variables included the age at surgery, age-adjusted Charlson co-morbidity score (CCM), Eastern Cooperative Oncology Group Performance Status score, and American Society of Anesthesiology (ASA) grade [23,24]. The CCM index has been used extensively in studies assessing post-operative outcomes in large datasets [25,26]. It is an accurate predictor of fitness for surgery and consistently correlates with the incidence of post-operative complications [24,27,28,29]. The CCM index was determined using an online calculator incorporating data from electronic patient records for each case [28]. Race and ethnicity data were not included, as these are not routinely collected as part of our centre’s surgical databases.

### 2.3. Definition of High and Low Surgeon–Anaesthetist Dyad Volume

All surgeon–anaesthetist dyad pairings were collated from the included cases. The median number of major resections performed per dyad was a total of *n* = 23 cases over the study period of 2014–2024. This median number of cases per dyad was arbitrarily chosen as a cut-off; therefore, dyads that performed *n* = 23 or more cases within this period were retrospectively classed as ‘high volume,’ while dyads that performed less than the median of *n* = 23 cases were classed as ‘low volume’. There was no difference in the calculated number of years’ experience between Consultant Anaesthetists in high- and low-volume dyads. All Consultant Surgeons were performing high-volume resectional surgery for upper GI cancer independently from the start of their independent consultant practice and throughout the study period.

### 2.4. Definition of Level of Risk

Previous research studies have stratified patients into high and low risk based on the median Charlson co-morbidity (CCM) score for all included patients [29]. This method of stratification has also been utilised in our study. This study’s median Charlson co-morbidity (CCM) score for all included patients was 5. Therefore, patients with CCM greater than or equal to 5 were classed as high risk. Patients with CCM less than this were defined as low risk.

### 2.5. Propensity Score Matching

Initially, all cases were analysed following allocation into one of four groups: high-volume surgeon–anaesthetist dyads and high-risk cases, high-volume dyads and low-risk cases, low-volume dyads and high-risk cases, and low-volume dyads and low-risk cases. However, there was considerable mismatch in the volume of cases performed per group. In particular, the majority of cases were performed by high-volume dyads, regardless of risk. We therefore carried out propensity score matching (PSM) accounting for age and CCM to adjust for this. All cases were divided into two groups as high-volume dyad or low-volume dyad, and PSM was performed for age and CCM. Scores were estimated via logistic regression, and cases were paired with one from each group according to propensity score in a 1:1 ratio without replacement. Successfully matched cases were divided into the following four groups again for analysis: high-volume dyad–high-risk cases, high-volume dyad–low-risk cases, low-volume dyad–high-risk cases, and low-volume dyad–low-risk cases. The low-volume dyad counts were identical before and after PSM; therefore, the low-volume cohort was retained in full as the matching anchor, with high-volume cases subsampled 1-to-1 against them.

### 2.6. Statistical Analyses

Descriptive and statistical analyses were conducted, and graphical representations were produced using RStudio (version 4.3.2, Boston, MA, USA). The extension package “MatchIT” was used to perform propensity score matching, along with the “tidyverse”, “dplyr”, and “ggplot2” packages. Chi-squared (χ^2^) or Fisher’s Exact Tests were used as appropriate. Logistic regression with interaction term analysis was performed between high-volume and low-volume dyads for propensity-score-matched cases producing odds ratios and 95% confidence intervals. Statistical significance was confirmed at *p* < 0.05. Data are expressed as median values unless otherwise specified.

## 3. Results

**Figure 1:**
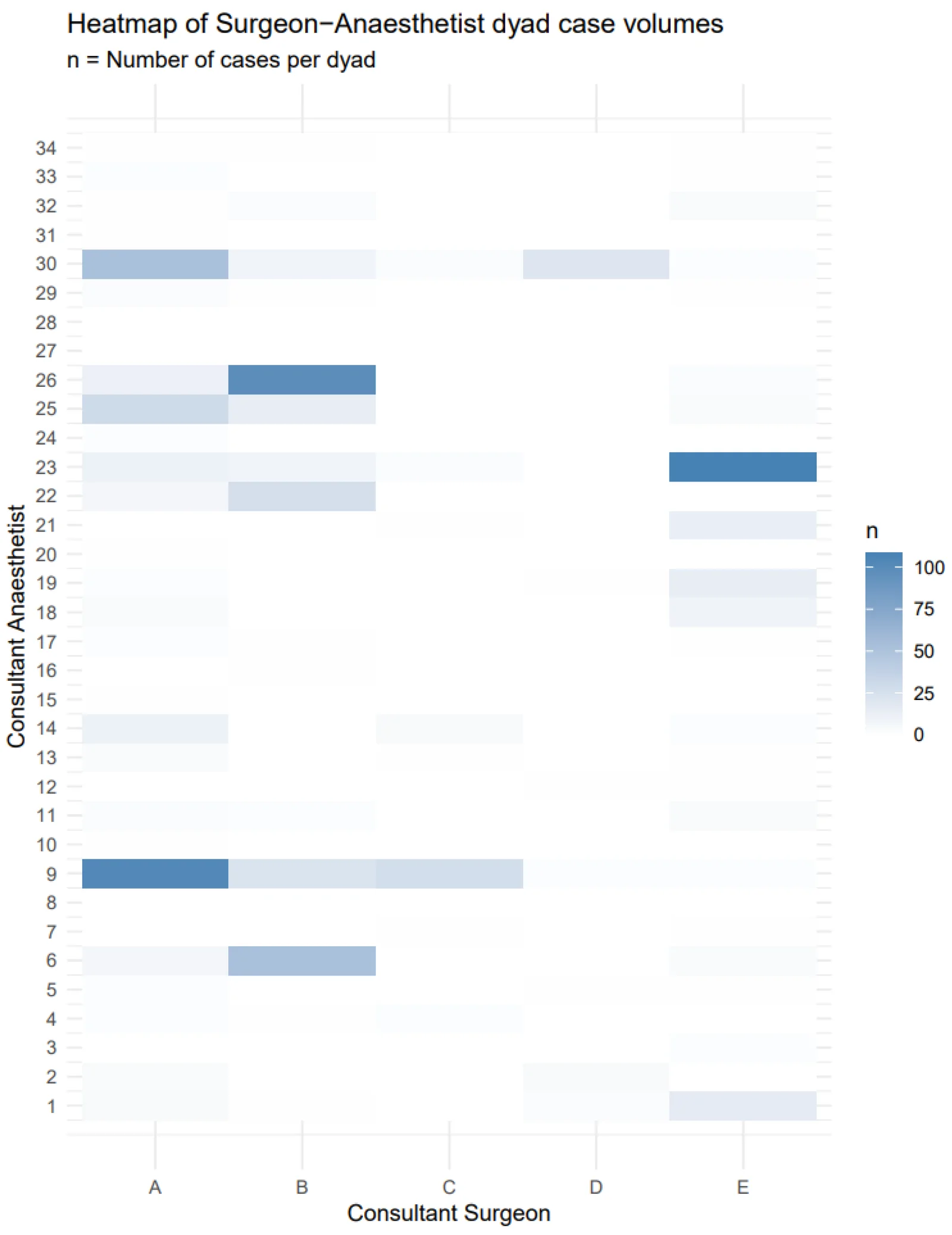
Heatmap of surgeon–anaesthetist dyads showing case volume (*n*) per dyad.

A total of *n* = 792 major upper gastrointestinal (GI) tract resections for primary oesophago–gastric (OG) and hepato–pancreato–biliary (HPB) carcinoma, and colorectal liver metastases (CRLMs) were performed by *n* = 5 Consultant Surgeons and *n* = 34 Consultant Anaesthetists between 2014 and 2024, of which there were a total of *n* = 77 dyad combinations (Table 1 and Table 2, Figure 1).

The highest number of cases performed by a single surgeon–anaesthetist dyad was *n* = 108 while the lowest was *n* = 1 (Figure 1). The most common OG and HPB procedures performed were a two-stage (Ivor–Lewis) oesophagectomy (*n* = 293, 37.0%) and pancreaticoduodenectomy (Whipple procedure) (*n* = 73, 9.22%), respectively (Table 1). The high-volume surgeon–anaesthetist dyads performed the majority of OG and HPB cases (*n* = 519, 65.5%) (Table 3).

## 4. Discussion

**Figure 2:**
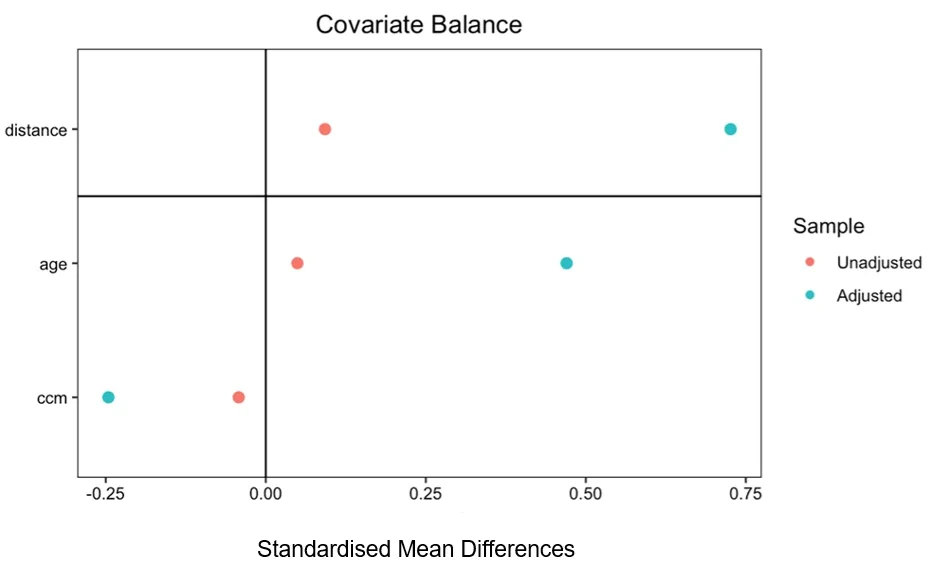
Covariate balance plot demonstrating the reduction in standardised mean differences from *n* = 792 unmatched (blue) cases to *n* = 546 matched (red) cases as a result of propensity score matching for age and Charlson co-morbidity score (CCM).

In this study, we examined the association between the volume of cases surgeon–anaesthetist dyads perform together and post-operative outcomes in complex resectional surgery for upper gastrointestinal (GI) and hepato–pancreato–biliary (HPB) malignancies over the last 10 years at our tertiary specialist cancer centre. After initial analysis, cases were propensity score matched for age and Charlson co-morbidity score in a 1:1 ratio to adjust for the larger proportion of patients that underwent surgery by high-volume dyads (Table 3, Figure 2).

We observed that high-volume dyads operating on high-risk cases had fewer complications overall (37.9%) compared to low-volume dyads operating on high-risk cases (42.8%) (Table 4a). This remained consistent following propensity score matching (35.2% vs. 42.8%), though neither comparison were statistically significant.

**Figure 3:**
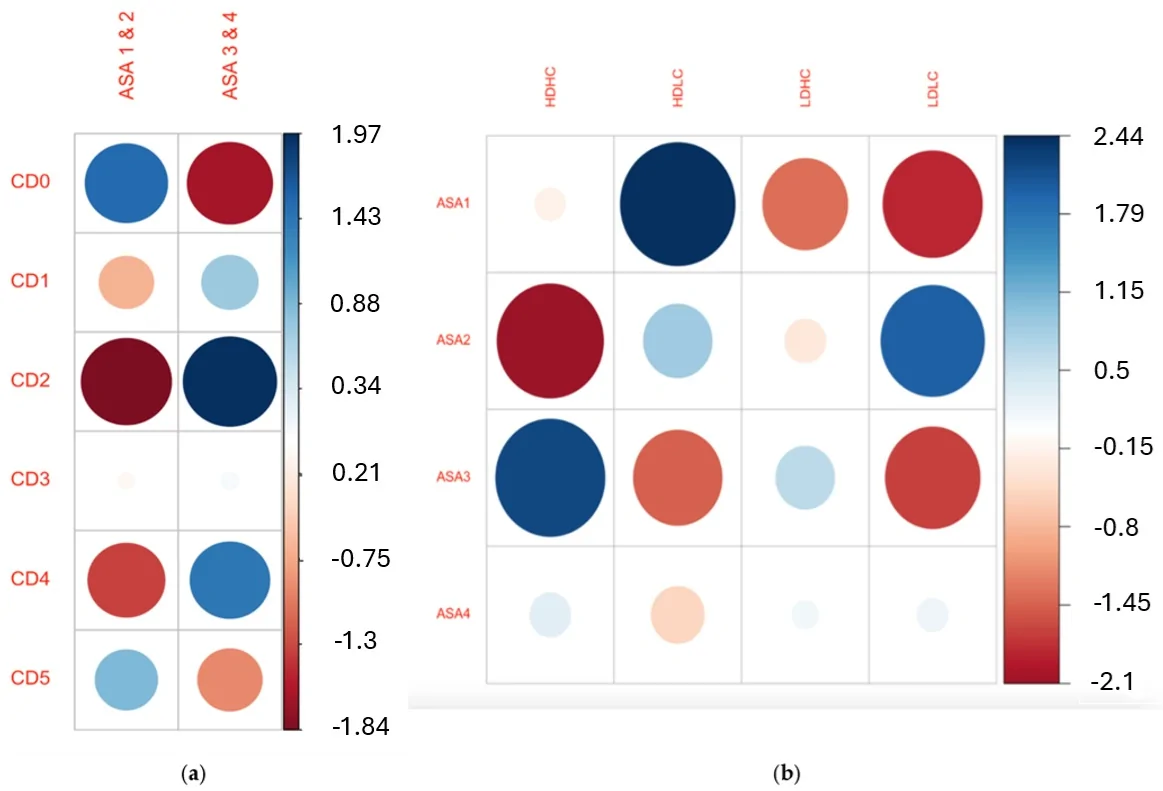
Heatmaps demonstrating that (**a**) higher-risk cases (ASA III and IV) were more likely to have Clavien–Dindo grade III and IV complications (*p* < 0.01) regardless of dyad volume, and that (**b**) high-volume dyads tended to operate on more patients of higher risk (ASA 3 and ASA 4) while low-volume dyads operated on patients of lower risk (ASA 2) (*p* < 0.001). Data were analysed using the Pearson chi-squared (χ^2^) test. The size of the circle is proportional to the contribution of the chi-squared (χ^2^) test result. Blue indicates a positive and red a negative association between (**a**) ASA grade and CD complications, and between (**b**) dyad volume, case co-morbidity, and ASA grade. CD: post-operative complications, as per the Clavien–Dindo classification. ASA: American Society of Anesthesiology. HD: high-volume dyad. LD: low-volume dyad. HC/LC: high/low co-morbidity score, as per the Charlson co-morbidity score (CCM).

High-volume dyads also had a lower incidence of Clavien–Dindo (CD) grade I (0%), II (23%), IIIA (3.83%), IIIB (1.15%), and V (1.92%) complications than low-volume dyads (1.38%, 25.5%, 5.52%, 1.38%, and 2.76%, respectively) (Table 4a) when operating on higher-risk patients. Following propensity score matching, it was identified that low-volume dyads operating on higher-risk patients had a greater incidence of CD grade I (1.38% vs. 0%), II (25.5% vs. 20.5%), III (6.90% vs. 4.92%), and V (2.76% vs. 1.64%) complications than high-volume dyads (Table 4b). Figure 3a indicates that higher-risk cases were more likely to have post-operative complications of greater severity than lower risk cases. Our data demonstrate that high-volume dyads are more likely to operate on these patients (Figure 3b) than lower-volume dyads (*p* < 0.001).

**Figure 4:**
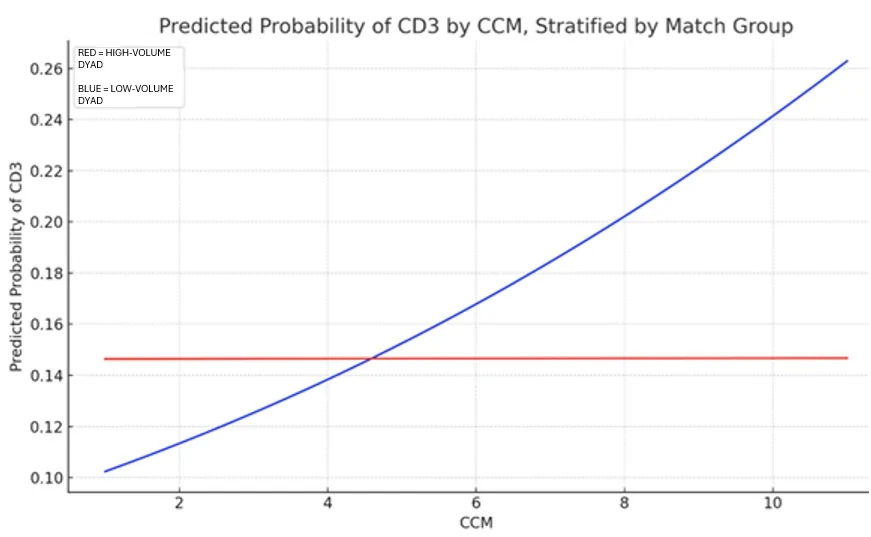
Logistic regression with interaction term analysis between high-volume and low-volume dyads for propensity-score-matched cases. Low-volume dyads (blue line) demonstrated an increased but non-significant trend in Clavien–Dindo grade III complications with an increase in Charlson co-morbidity score (CCM) (OR 1.121, 95% CI: 0.906–1.386, *p* = 0.224), whereas no change was demonstrated in high-volume dyads (red line) with an increase in CCM (OR 1.000 95% CI: 0.759–1.319, *p* = 0.999). OR: odds ratio, CI: confidence interval.

Furthermore, we observed a trend in which high-volume dyads had a lower incidence of grade IIIA (3.10%), IIIB (1.94%), and IVB (1.16%) complications than low-volume dyads operating on low-risk cases (3.91%, 2.34%, and 2.34%, respectively) (Table 4a), although this did not reach statistical significance (*p* = 0.669). In the propensity-matched cohort, low-volume dyads had a higher incidence of CD grade III complications, regardless of risk (6.9% vs. 4.92% and 6.25% vs. 5.3%, *p* = 0.829, Table 4b). We therefore examined this further by performing logistic regression with interaction term analysis (Figure 4). This revealed that the probability of a CD grade III complication (procedures under local anaesthetic, sedation, or general anaesthetic, including a return to theatre) could rise with an increase in CCM for low-volume dyads (OR 1.121, *p* = 0.224), although this did not reach statistical significance. These findings reinforce the importance of careful case selection for surgeon–anaesthetist dyads when scheduling major complex resectional surgery for upper GI and HPB malignancies.

When comparing total inpatient lengths of stay (LoS) between the four cohorts, we identified that high-volume dyads performing HPB procedures on low-risk patients had a lower median total inpatient LoS (9.5 days) compared to low-volume dyads (12.5 days) (Supplementary Tables S3 and S4). There were no significant differences in the critical care unit (CCU) LoS when comparing the four cohorts (Supplementary Tables S1–S4). We observed that the greater the volume of procedures surgeons and anaesthetists perform together, the lower the rates of recorded complications with equivalence in post-operative outcomes, including CCU LoS, between low- and high-risk patients. This lower rate of recorded complications may have an impact on further treatment options, including those requiring adjuvant therapy.

In an operating theatre environment, clear communication and mutual understanding between the surgeon and anaesthetist are key. This becomes particularly crucial in the event of intra-operative emergencies, including major haemorrhage, where time-critical decision-making may have a significant impact on patient outcomes [19,30]. Resectional surgery for UGI malignancy is complex, and close attention must be paid to patients’ intra-operative physiology. Examples of this include the change in ventilatory requirements for one-lung ventilation (OLV) and the need for prudent fluid management during two- or three-stage oesophagectomy, as well as intra-operative central venous monitoring with synchronous fluid management during hepatic resection [19,31,32]. Fluid management preferences vary between surgeons, but this may have a significant influence when resecting malignant liver lesions and identifying bleeding [31]. A two-stage oesophagectomy involves operating in the thorax and abdomen, as well as in the neck in three-stage procedures. Each phase has differing fluid needs: larger volumes are required during the abdominal phase, but a more restrictive approach is needed in the thoracic phase [33]. Therefore, judicious management of fluid administration and monitoring is required, especially as this can influence the risk of post-operative pulmonary complications [32,34,35]. Expertise in managing double-lumen tubes may be achieved through performing a greater volume of cases, with fibreoptic bronchoscopy as an important additional skill to augment airway assessment [32,36]. This is reflected in the scientific literature, which indicates that poorly managed lung ventilation is associated with prolonged post-operative intubation, increased risks of post-operative pneumonia, and greater critical care and total inpatient LoS [34,35]. A further example of the importance of collaboration is in patient positioning. At our centre, patients are positioned into the left lateral decubitus position for the open thoracic phase of a two-stage oesophagectomy. Inadequate patient positioning can affect OLV and risk pulmonary complications. In addition, appropriate positioning is required to obtain sufficient exposure of the intra-thoracic anatomy following thoracotomy to enable a radical resection with lymphadenectomy [33,37,38]. This reiterates the importance of greater collaboration through clear communication and familiarity between the surgeon and anaesthetist, which we suggest can be developed through a higher volume of surgical practice together.

Significant research has been conducted on non-technical skills in the operating theatre and their impact on both minor intra-operative incidents, including equipment delays, and major incidents impacting patient safety [39]. Although it is challenging to measure the ability of non-technical skills and quality of teamwork directly, it has been demonstrated that poor leadership, communication, and situational awareness were noted to correlate directly with adverse intra-operative incidents [40]. Failure to communicate and a lack of situational awareness may be corrected through greater familiarity between team members, achievable by performing a greater volume of cases together. The non-technical skills for surgeons and anaesthetists, termed ‘NOTSS’ and ‘ANTS’, respectively, are taxonomies developed to allow for the assessment and development of interpersonal skills within and between both groups [39,40,41,42]. It may be argued that surgeons and anaesthetists are at the core of operating theatre dynamics and are pivotal in ensuring optimum patient outcomes. As a result, non-technical skills could be embedded into the senior years of postgraduate surgical and anaesthetic training, enabling future Consultant Surgeons and Consultant Anaesthetists to be able to lead and work effectively together.

The team in the operating theatre extends far beyond the Consultant Surgeon and Anaesthetist and includes scrub staff and operating department practitioners. Future research could extend the ‘dyad’ to a ‘polyad’ of health professionals to ascertain whether consistency of the polyad is associated with better post-operative outcomes and theatre efficiency.

The surgeon and anaesthetist team has a multi-faceted role in the prehabilitation of patients undergoing major upper GI and HPB cancer surgery. A multi-disciplinary approach could optimise medical co-morbidities, exercise tolerance prior to surgery, and patient understanding of the implications of surgery and anaesthesia. At our centre, all patients undergoing resectional surgery for carcinoma of the oesophagus or gastro–oesophageal junction (GOJ) and selected high-risk patients undergoing pancreatectomy, pancreaticoduodenectomy, or major liver resection are discussed at a fortnightly multi-disciplinary pre-operative meeting attended by their responsible Consultant Surgeon and Consultant Anaesthetist, as well as their dieticians, physiotherapists, pain specialists, and psychologists. Patients are selected for this meeting based on surgeons’ clinical assessment of the patient in clinic, confirmation of resectability, and review of their co-morbidities. This provides an open forum to discuss patients’ co-morbidities and identify those that may benefit from additional investigations, including cardiopulmonary exercise testing to inform treatment decisions and guide personalised physical prehabilitation programmes to optimise patients undergoing major surgery. Regular discussions of complex cases in this setting ensures alignment of the anaesthetic and surgical goals for each case. As well as enhancing familiarity between the surgeon and anaesthetist, this serves as a way of increasing trust, sharing knowledge, and enhancing cooperation within the dyad, which may influence patient outcomes. An additional approach to enhancing multi-disciplinary teamworking could include weekly pre-operative planning meetings with dedicated Gastrointestinal Radiologists to review imaging of upcoming cases. This could improve surgical planning and highlight potential intra-operative challenges, which may lead to improved post-operative outcomes and provide a teaching opportunity for trainees. Scope for a future study may therefore include assessing the impact of consistently paired teams on training in surgery and anaesthesia; that is, whether working with the same Consultant Surgeon and Consultant Anaesthetist may be associated with improved development of technical skills among trainees aspiring to a career in major upper GI cancer surgery or anaesthesia.

In patients undergoing surgery for oesophago–gastric (OG) carcinoma, an uneventful recovery with clear oncological resection margins enables the prompt commencement of adjuvant systemic therapy [3]. We suggest that the combination of high rates of peri-operative oncological treatment completion with optimal multi-disciplinary pre- and peri-operative management for surgery are critical in improving patient outcomes, including overall survival [32]. We present a multi-modal approach to improving outcomes from major UGI surgery, with the surgeon–anaesthetist working relationship a key foundation of this.

Our data suggest that high-volume dyads could safely operate on more complex patients with UGI malignancies, as described by their ASA score, without an increase in CCU or total inpatient LoS. This was applicable for both OG and HPB resections in our centre. Therefore, it may be extrapolated to suggest new surgeon–anaesthetist dyads may benefit from operating on lower-risk cases together, allowing time for relationship building and teamwork. A stepwise approach may be adopted to undertaking more challenging or co-morbid patients after developing initial experience. This reiterates that the more cases a surgeon–anaesthetist dyad performs, the more potential there is for better post-operative outcomes for patients undergoing surgical resection for UGI cancers.

## 5. Limitations

There have been considerable changes in oncological therapies, surgical technology, and peri-operative care for UGI tract cancer over the ten years of data we have analysed. Concurrently, surgeons’ and anaesthetists’ clinical practices and skills may have evolved over this period as more experience was gained.

Examples of changes in oncological therapies and surgical technology are potential confounding variables and include the wider uptake of peri-operative FLOT (5-fluorouracil, leucovorin, oxaliplatin and docetaxel) in oesophago–gastric (OG) adenocarcinoma, adjuvant immunotherapy in oesophageal or GOJ cancer, and robotic surgery and neoadjuvant chemoradiotherapy in resectable and borderline resectable pancreatic cancer, or upfront surgery where indicated [3,10,11,12,13]. Our data include both OG and HPB cases, which have varying treatment paradigms within these cohorts. Although subgroup analyses were conducted separately for OG and HPB resections, as well as for CRLMs, no significant findings were elicited.

Further confounding factors include the introduction of prehabilitation programmes and specialist peri-operative care for patients with higher co-morbidity undergoing upper GI cancer surgery at our centre. Since their implementation, our centre has noted a decrease in the length of stay, both in the CCU and in the hospital overall. In this study, we have shown that higher-volume dyads were more likely to operate on more complex cases as determined by CCM score. This introduces an element of procedure allocation bias that leads to clustering, as certain surgeons and anaesthetists may wish to work together more exclusively and therefore may schedule more complex cases to operate on together.

The confounding factors discussed considerably influence the clinical and oncological outcomes of patients undergoing resectional surgery but are also highly heterogenous, which makes adjusting for these variables challenging. However, the high volume of cases included within this study and propensity score matching analyses reduce the risk of bias introduced by heterogeneity.

Where previous national-scale studies have examined the impact of dyad volume on post-operative outcomes, they have acknowledged that certain factors, including the type of centre performing the operations (academic or community hospital) and subsequent variations in institutional peri-operative management pathways, can influence post-operative outcomes [18]. Our study is a retrospective analysis from a single specialist cancer centre where elective, major resectional procedures comprise the bulk of the workload. However, there is a greater degree of consistency in peri-operativee management protocols, which may also address some of the challenges of heterogeneity within the study cohorts.

The centralisation of services in the UK has extended to trauma, complex benign surgery, and OG emergencies, including Boerhaave’s syndrome. This may require the expertise and skillsets of the same surgeons, anaesthetists, and intensivists that manage patients undergoing resectional surgery for UGI tract cancer. Consequently, this variation in elective and emergency workload, and the subsequent impact on work schedules, may limit the volume of resections performed by dyads at other units.

## 6. Conclusions

This study suggests that the greater the volume of cases a surgeon–anaesthetist dyad performs, the more likely they are to operate on higher-risk patients for whom resectional surgery is indicated for UGI tract malignancy. A higher dyad volume was associated with a non-significant trend towards fewer post-operative complications. However, these findings require confirmation in larger, multi-centre datasets. A potential implication of this study is to take dyad volume into consideration when scheduling complex resectional upper GI cancer surgery.

## Figures and Tables

**Table 1:** Most common major oesophago–gastric and hepato–pancreato–biliary resections from 2014 to 2024.

Procedure	*n*	% ^a^
Two-stage oesophagectomy	293	37
Total gastrectomy	112	14.1
Subtotal gastrectomy	90	11.4
Pancreatoduodenectomy (Whipple procedure)	73	9.22
Major liver resection	65	8.21
Left thoraco–abdominal oesophagogastrectomy	45	5.68
Pylorus-preserving pancreatodudodenectomy	34	4.29
Extended total gastrectomy	34	4.29
Three-stage oesophagectomy	10	1.26
Distal pancreatectomy	10	1.26
Total pancreatectomy	9	1.14
Central hepatectomy	7	0.883
Mini mesohepatectomy	6	0.758

^a^ Percentage of all *n* = 792 cases included.

**Table 2:** Summary of dyad case volumes and risks—unmatched cases.

	Number of Cases Performed During Study Period (*n*) (% ^a^)
High-volume dyad, high risk	261 (33.0%)
Low-volume dyad, high risk	145 (18.3%)
High-volume dyad, low risk	258 (32.6%)
Low-volume dyad, low risk	128 (16.2%)
Total	792

High-volume dyad: ≥23 cases. Low-volume dyad: <23 cases. High risk: Charlson co-morbidity score ≥5. Low risk: Charlson co-morbidity score <5. ^a^ Percentage of all *n* = 792 cases performed during study period.

**Table 3:** Summary of dyad case volumes and risk stratified by specialty following propensity score matching for age and Charlson co-morbidity score.

	Oesophago–Gastric (*n*) (% ^a^)	HPB (*n*) (% ^a^)	Total (*n*) (% ^a^)
High-volume dyad, high risk	106 (19.4%)	16 (2.93%)	122 (22.3%)
Low-volume dyad, high risk	86 (15.8%)	59 (10.8%)	145 (26.6%)
High-volume dyad, low risk	138 (25.3%)	13 (2.38%)	151 (27.8%)
Low-volume dyad, low risk	97 (17.8%)	31 (5.68%)	128 (23.4%)
Total	427 (78.2%)	119 (21.8%)	546

HPB: hepato–pancreato–biliary. High-volume dyad: ≥23 cases. Low-volume dyad: <23 cases. High risk: Charlson co-morbidity score ≥5. Low risk: Charlson co-morbidity score <5. ^a^ Percentage of *n* = 546 propensity-score-matched cases.

**Table 4:** Pooled Clavien–Dindo complications stratified by dyad volume and risk for (a) unmatched (*n* = 792 cases, *p* = 0.669) and (b) propensity-score-matched cases (*n* = 546 cases, *p* = 0.829). Data were analysed using the Pearson chi-squared (χ^2^) test. High-volume dyad: ≥23 cases. Low-volume dyad: <23 cases. High risk: Charlson co-morbidity score ≥5. Low risk: Charlson co-morbidity score <5.

**(a)**														
		**Clavien–Dindo Complications (%, ** ***n*** **)**												
		0	I		II		IIIA		IIIB	IVA		IVB		V
High-volume dyad, high risk (*n* = 261)		62.1 (162)	0 (0)		23.0 (60)		3.83 (10)		1.15 (3)	6.51 (17)		1.53 (4)		1.92 (5)
Low-volume dyad, high risk (*n* = 145)		57.2 (83)	1.38 (2)		25.5 (37)		5.52 (8)		1.38 (2)	6.21 (9)		0 (0)		2.76 (4)
High-volume dyad, low risk (*n* = 258)		65.1 (168)	1.16 (3)		20.2 (52)		3.10 (8)		1.94 (5)	6.20 (16)		1.16 (3)		1.16 (3)
Low-volume dyad, low risk (*n* = 128)		67.2 (86)	0.781 (1)		18.0 (23)		3.91 (5)		2.34 (3)	5.47 (7)		2.34 (3)		0 (0)
**(b)**														
	**Clavien–Dindo Complications (%, ** ***n*** **)**													
	0			I		II		III			IV		V	
High-volume dyad, high risk (*n* = 122)	64.8 (79)			0 (0)		20.5 (25)		4.92 (6)			8.20 (10)		1.64 (2)	
Low-volume dyad, high risk (*n* = 145)	57.2 (83)			1.38 (2)		25.5 (37)		6.90 (10)			6.21 (9)		2.76 (4)	
High-volume dyad, low risk (*n* = 151)	65.6 (99)			0.662 (1)		19.2 (29)		5.30 (8)			7.95 (12)		1.32 (2)	
Low-volume dyad, low risk (*n* = 128)	67.2 (86)			0.781 (1)		18.0 (23)		6.25 (8)			7.81 (10)		0 (0)	

## Data Availability

Data can be obtained by contacting the corresponding author.

## Supplementary Materials

The following supporting information can be downloaded at: https://www.mdpi.com/article/10.3390/cancers18162621/s1, Table S1. Outcomes of high-volume dyads operating on high-risk cases; Table S2. Outcome of low-volume dyads operating on high-risk cases; Table S3. Outcomes of high-volume dyads operating on low-risk cases; Table S4. Outcomes of low-volume dyads operating on low-risk cases.

## Abbreviations

The following abbreviations are used in this manuscript:

ASA	American Society of Anesthesiology
CD	Clavien–Dindo classification
CRLM	Colorectal liver metastasis
GI	Gastrointestinal
GOJ	Gastro–oesophageal junction
HPB	Hepato–pancreato–biliary
OG	Oesophago–gastric
UGI	Upper gastrointestinal

## References

[1] Cooper J.B. (2018). Critical Role of the Surgeon-Anesthesiologist Relationship for Patient Safety. Anesthesiology.

[2] Schmidt C.M., Turrini O., Parikh P., House M.G., Zyromski N.J., Nakeeb A., Howard T.J., Pitt H.A., Lillemoe K.D. (2010). Effect of Hospital Volume, Surgeon Experience, and Surgeon Volume on Patient Outcomes After Pancreaticoduodenectomy. Arch. Surg..

[3] Patel P.H., Patel N.M., Doyle J.P., Patel H.K., Alhasan Y., Luangsomboon A., Petrou N., Bhogal R.H., Kumar S., Chaudry M.A. (2024). Circumferential resection margin rates in esophageal cancer resection- oncological equivalency and comparable clinical outcomes between open versus minimally invasive techniques: A retrospective cohort study. Int. J. Surg..

[4] Birkmeyer J.D., Stukel T.A., Siewers A.E., Goodney P.P., Wennberg D.E., Lucas F.L. (2003). Surgeon volume and operative mortality in the United States. N. Engl. J. Med..

[5] Glasgow R.E., Showstack J.A., Katz P.P., Corvera C.U., Warren R.S., Mulvihill S.J. (1999). The Relationship Between Hospital Volume and Outcomes of Hepatic Resection for Hepatocellular Carcinoma. Arch. Surg..

[6] Hendricks A., Diers J., Baum P., Weibel S., Kastner C., Müller S., Lock J.F., Köller F., Meybohm P., Kranke P. (2021). Systematic review and meta-analysis on volume-outcome relationship of abdominal surgical procedures in Germany. Int. J. Surg..

[7] Levaillant M., Marcilly R., Levaillant L., Michel P., Hamel-Broza J.-F., Vallet B., Lamer A. (2021). Assessing the hospital volume-outcome relationship in surgery: A scoping review. BMC Med. Res. Methodol..

[8] Clavien P.A., Sanabria J.R., Strasberg S.M. (1992). Proposed classification of complications of surgery with examples of utility in cholecystectomy. Surgery.

[9] Hallet J., Jerath A., Turgeon A.F., McIsaac D.I., Eskander A., Zuckerman J., Zuk V., Sohail S., Darling G.E., Dharma C. (2021). Association Between Anesthesiologist Volume and Short-term Outcomes in Complex Gastrointestinal Cancer Surgery. JAMA Surg..

[10] Doyle J.P., Patel P.H., Doran S.L.F., Jiao L.R., Cunningham D., Nicol D., Mavroeidis V.K., Allum W.H., Chaudry A.M., Bhogal R.H. (2023). The Cancer Hub Approach for Upper Gastrointestinal Surgery During COVID-19 Pandemic: Outcomes from a UK Cancer Centre. Ann. Surg. Oncol..

[11] Pugalenthi A., Protic M., Gonen M., Kingham T.P., D’Angelica M.I., Dematteo R.P., Fong Y., Jarnagin W.R., Allen P.J. (2016). Postoperative complications and overall survival after pancreaticoduodenectomy for pancreatic ductal adenocarcinoma. J. Surg. Oncol..

[12] Kamaleddine I., Hendricks A., Popova M., Schafmeyer C. (2022). Adequate Management of Postoperative Complications after Esophagectomy: A Cornerstone for a Positive Outcome. Cancers.

[13] Melnychuk M., Vindrola-Padros C., Aitchison M., Clarke C.S., Fulop N.J., Levermore C., Maddineni S.B., Moore C.M., Mughal M.M., Perry C. (2018). Centralising specialist cancer surgery services in England: Survey of factors that matter to patients and carers and health professionals. BMC Cancer.

[14] Lambert J.E., Hayes L.D., Keegan T.J., Subar D.A., Gaffney C.J. (2021). The Impact of Prehabilitation on Patient Outcomes in Hepatobiliary, Colorectal, and Upper Gastrointestinal Cancer Surgery: A PRISMA-Accordant Meta-analysis. Ann. Surg..

[15] Thomas G., Tahir M.R., Bongers B.C., Kallen V.L., Slooter G.D., van Meeteren N.L. (2019). Prehabilitation before major intra-abdominal cancer surgery. Eur. J. Anaesthesiol..

[16] Joung R.H.-S., Bilimoria K.Y., Merkow R.P. (2021). Behind the Curtain: Impact of Anesthesia Volume on Outcomes. JAMA Surg..

[17] Togioka B.M., Mayo S.C. (2023). Surgeon-Anesthesiologist Dyad Familiarity-What Are the Unintended Consequences. JAMA Surg..

[18] Hallet J., Sutradhar R., Jerath A., d’Empaire P.P., Carrier F.M., Turgeon A.F., McIsaac D.I., Idestrup C., Lorello G., Flexman A. (2023). Association Between Familiarity of the Surgeon-Anaesthesiologist Dyad and Postoperative Patient Outcomes for Complex Gastrointestinal Cancer Surgery. JAMA Surg..

[19] Weldon S.-M., Korkiakangas T., Bezemer J., Kneebone R. (2013). Communication in the operating theatre. Br. J. Surg..

[20] Mills P., Neily J., Dunn E. (2008). Teamwork and communication in surgical teams: Implications for patient safety. J. Am. Coll. Surg..

[21] Merry A.F., Weller J.M. (2021). Communication and team function affect patient outcomes in anaesthesia: Getting the message across. Br. J. Anaesth..

[22] Von Elm E., Altman D.G., Egger M., Pocock S.J., Gøtzsche P.C., Vandenbroucke J.P. (2007). STROBE Initiative. The Strengthening the Reporting of Observational Studies in Epidemiology (STROBE) statement: Guidelines for reporting observational studies. Lancet.

[23] Charlson M.E., Pompei P., Ales K.L., MacKenzie C.R. (1987). A new method of classifying prognostic comorbidity in longitudinal studies: Development and validation. J. Chronic Dis..

[24] Quan H., Li B., Couris C.M., Fushimi K., Graham P., Hider P., Januel J.-M., Sundararajan V. (2011). Updating and validating the Charlson comorbidity index and score for risk adjustment in hospital discharge abstracts using data from 6 countries. Am. J. Epidemiol..

[25] Lagergren J., Bottai M., Santoni G. (2021). Patient Age and Survival After Surgery for Esophageal Cancer. Ann. Surg. Oncol..

[26] Dias-Santos D., Ferrone C.R., Zheng H., Lillemoe K.D., Fernández-Del Castillo C. (2015). The Charlson age comorbidity index predicts early mortality after surgery for pancreatic cancer. Surgery.

[27] Quan H., Sundararajan V., Halfon P., Fong A., Burnand B., Luthi J.-C., Saunders L.D., Beck C.A., Feasby T.E., Ghali W.A. (2005). Coding algorithms for defining comorbidities in ICD-9-CM and ICD-10 administrative data. Med. Care.

[28] Brusselaers N., Lagergren J. (2017). The Charlson Comorbidity Index in Registry-based Research. Methods Inf. Med..

[29] Kim S., Park J., Kwon J.-H., Oh A.R., Gook J., Yang K., Choi J.-H., Kim K., Sung J.D., Ahn J. (2021). The Charlson Comorbidity Index is associated with risk of 30-day mortality in patients with myocardial injury after non-cardiac surgery. Sci. Rep..

[30] Sydor D.T., Bould M.D., Naik V.N., Burjorjee J., Arzola C., Hayter M., Friedman Z. (2013). Challenging authority during a life-threatening crisis: The effect of operating theatre hierarchy. Br. J. Anaesth..

[31] Yoshino O., Perini M.V., Christophi C., Weinberg L. (2017). Perioperative fluid management in major hepatic resection: An integrative review. Hepatobiliary Pancreat. Dis. Int..

[32] Deana C., Vetrugno L., Bignami E., Bassi F. (2021). Peri-operative approach to esophagectomy: A narrative review from the anesthesiological standpoint. J. Thorac. Dis..

[33] Bonavina L., Asti E., Sironi A., Bernardi D., Aiolfi A. (2017). Hybrid and total minimally invasive esophagectomy: How I do it. J. Thorac. Dis..

[34] Avendano C.E., Flume P.A., Silvestri G.A., King L.B., Reed C.E. (2002). Pulmonary complications after esophagectomy. Ann. Thorac. Surg..

[35] Zingg U., Smithers B.M., Gotley D.C., Smith G., Aly A., Clough A., Esterman A.J., Jamieson G.G., Watson D.I. (2011). Factors associated with postoperative pulmonary morbidity after esophagectomy for cancer. Ann. Surg. Oncol..

[36] Campos J.H., Hallam E.A., Ueda K. (2011). Training in placement of the left-sided double-lumen tube among non-thoracic anaesthesiologists: Intubation model simulator versus computer-based digital video disc, a randomised controlled trial. Eur. J. Anaesthesiol..

[37] Thammineedi S.R., Patnaik S.C., Nusrath S. (2020). Minimal Invasive Esophagectomy—A New Dawn of Esophageal Surgery. Indian J. Surg. Oncol..

[38] Schizas D., Papaconstantinou D., Krompa A., Athanasiou A., Triantafyllou T., Tsekrekos A., Ruurda J.P., Rouvelas I. (2022). Minimally invasive oesophagectomy in the prone versus lateral decubitus position: A systematic review and meta-analysis. Dis. Esophagus.

[39] Paterson-Brown S., Youngson G., McIlhenny C., Maran N., Flin R., Yule S. (2019). Raising awareness of non-technical skills in operating theatres. BMJ.

[40] Siu J., Maran N., Paterson-Brown S. (2016). Observation of behavioural markers of non-technical skills in the operating room and their relationship to intra-operative incidents. Surgeon.

[41] Yule S., Flin R., Paterson-Brown S., Maran N., Rowley D. (2006). Development of a rating system for surgeons’ non-technical skills. Med. Educ..

[42] Flin R., Patey R., Glavin R., Maran N. (2010). Anaesthetists; non-technical skills. Br. J. Anaesth..

