# Supplementary material for: Impact of Greater Surgeon–Anaesthetist Familiarity on Post-Operative Outcomes in Upper Gastrointestinal Cancer Surgery: A 10-Year UK Tertiary Cancer Centre Experience
Source: Cancers (Basel). 2026 Aug 14;18(16):2621. doi: 10.3390/cancers18162621 (PMC13510901; doi:10.3390/cancers18162621)
Supplement: Supplementary file 1 [file cancers-18-02621-s001.zip › cancers-4361422-supplementary.pdf]

Table S1. Outcomes of high volume dyads operating on high risk cases.

	OG		HPB		COMBINED	
	**n**	**%**	**n**	**%**	**n**	**%**
**Male**	139	77.7	49	59.8	188	**72.0**
**Female**	40	22.3	33	40.2	73	**28.0**
**TOTAL**	179		82		261	

**ASA**	**n**	**%**	**n**	**%**	**n**	**%**
**1**	5	2.79	1	1.23	6	2.30
**2**	62	34.6	33	40.2	95	36.4
**3**	100	55.9	29	35.4	129	49.4
**4**	0	0	2	2.44	2	0.766
**Not recorded**	12	6.70	17	20.7	29	11.1

	**Median (IQR)**	**Median (IQR)**
**ECOG performance status**	1(1)	1 (1)
**Age at surgery (years)**	73 (6.45)	70 (14)

**Procedure**	**n**	**%**	**Procedure**	**n**	**%**
2-stage oesophagectomy	99	55.3	Whipple procedure	31	37.8
Subtotal gastrectomy	31	17.3	Major Liver Resection	26	31.7
Total gastrectomy	29	16.2	Pylorus-preserving pancreatoduodenectomy (PPPD)	7	8.54
LTA oesophagogastrecto my	5	2.79	Central hepatectomy	4	4.88
Extended total gastrectomy	7	3.91	Total pancreatectomy	2	2.44
3-stage oesophagectomy	4	2.23	Mini-mesohepatectomy	3	3.66
Completion gastrectomy	2	1.12	Left hemihepatectomy	2	2.44
Distal oesophagectomy + extended total gastrectomy	1	0.559	Right hemihepatectomy	2	2.44
Completion extended total gastrectomy	1	0.559	Extended distal pancreatectomy + splenectomy	2	2.44
**TOTAL**	179		Radical antegrade modular pancreatosplenectomy (RAMPS)	2	2.44
			Posterior sectionectomy	1	1.22
			**TOTAL**	82	

**Clavien-Dindo**	**n**	**%**	**n**	**%**	**n**	**%**

None	111	62.0	51	62.3	162	62.1
I	0	0	0	0	0	0
II	36	52.9	24	77.4	60	60.1
IIIA	6	8.82	4	12.9	10	10.1
IIIB	2	2.94	1	3.23	3	3.03
IVA	16	23.5	1	3.23	17	17.2
IVB	4	5.88	0	0	4	4.04
V	4	5.88	1	3.23	5	5.05
Total complications	68	38.0	31	37.8	99	37.9
	**Median (IQR)**		**Median (IQR)**	**Median (IQR)**		
**CCU LoS (days)**	4 (4)		2 (3)	3 (3)		
**Total IP LoS (days)**	13 (5)		11 (9)	13 (7)		
	n	%	n	%	n	%
**30-day mortality**	1	0.559	0	0	1	0.383
**90-day mortality**	4	2.23	1	1.22	5	1.92
	**n**	**%**	**n**	**%**	**n**	
**R0**	161	89.9	59	72.0	220	84.3
**R1**	16	8.94	14	17.1	30	11.5
**Not specified**	2	1.12	9	11.0	11	4.21

OG; Oesophago-gastric, HPB; Hepato-pancreato-biliary, LTA; Left Thoraco-abdominal, CCU; Critical Care Unit, IP; Inpatient, LoS; Length of Stay, R0; resection margins clear of microscopic evidence of carcinoma, R1; resection margins positive for microscopic evidence of cancer.

Table S2. Outcome of low volume dyads operating on high risk cases.

	**OG**		**HPB**		**COMBINED**	
	**n**	**%**	**n**	**%**	**n**	**%**
**Male**	63	73.3	29	49.2	92	63.4
**Female**	23	26.7	30	50.8	53	36.6
**TOTAL**	86		59		145	
**ASA**	**n**	**%**	**n**	**%**	**n**	**%**
**1**	1	1.16	0	0	1	0.690
**2**	37	43.0	25	42.4	62	42.8
**3**	40	46.5	23	39.0	63	43.4
**4**	1	1.16	0	0	1	0.690
**Not recorded**	7	8.14	11	18.6	18	12.4
	**Median (IQR)**		**Median (IQR)**			
**ECOG performance status**	1 (1)		1 (1)			
**Age at surgery (years)**	73 (8)		71 (22.5)			

Procedure	n	%	Procedure	n	%
2-stage oesophagectomy	34	39.5	Major Liver Resection	19	32.2
Subtotal gastrectomy	22	25.6	Whipple procedure	11	18.6
Total gastrectomy	13	15.1	Pylorus-preserving pancreaticoduodenectomy	10	16.9
LTA oesophagogastrectomy	7	8.14	Total pancreatectomy	4	6.78
Extended total gastrectomy	6	6.98	Distal pancreatectomy	4	6.78
Completion gastrectomy	1	1.16	Mini-mesohepatectomy	2	3.39
Proximal gastrectomy	1	1.16	Right hemihepatectomy	3	5.08
Distal oesophagectomy and proximal gastrectomy	1	1.16	Right posterior sectionectomy	2	3.39
Transhiatal oesophagectomy	1	1.16	Extended distal pancreatectomy	2	3.39
**TOTAL**	86		Central hepatectomy	1	1.69
			Extended left hemihepatectomy	1	1.69
			**TOTAL**	59	

Clavien-Dindo	n	%	n	%	n	%
None	54	62.8	29	49.2	83	55.7
I	0	0	2	6.67	2	3.23
II	18	56.3	19	63.3	37	59.7
IIIA	3	9.38	5	16.7	8	12.9
IIIB	2	6.25	0	0	2	3.23
IVA	6	18.8	3	10	9	14.5
IVB	0	0	0	0	0	0
V	3	9.38	1	3.33	4	6.45
Total complications	32	37.2	30	50.8	62	42.8

	Median (IQR)	Median (IQR)	Median (IQR)
**CCU LoS (days)**	4 (5)	2 (2)	3 (4)
**Total IP LoS (days)**	13 (6)	11 (9)	13 (8.25)

	n	%	n	%	n	%
**30-day mortality**	1	1.16	0	0	1	0.690
**90-day mortality**	2	2.33	1	1.69	3	2.07

	n	%	n	%	n	%
**R0**	74	86.0	50	84.7	124	85.5
**R1**	11	12.8	4	6.78	15	10.3

**Not specified**	1	1.16	5	8.47	6	4.14

OG; Oesophago-gastric, HPB; Hepato-pancreato-biliary, LTA; Left Thoraco-abdominal, CCU; Critical Care Unit, IP; Inpatient, LoS; Length of Stay, R0; resection margins clear of microscopic evidence of carcinoma, R1; resection margins positive for microscopic evidence of cancer.

Table S3. Outcomes of high volume dyads operating on low risk cases.

	**OG**		**HPB**		**COMBINED**	
	**n**	**%**	**n**	**%**	n	%
**Male**	169		14	53.8	183	
**Female**	63		12	46.2	75	
**TOTAL**	232		26		258	

**ASA**	**n**	**%**	**n**	**%**	n	%
**1**	9		2	7.69	11	4.26
**2**	123		9	34.6	132	51.2
**3**	85		10	38.5	95	36.8
**4**	1		0	0	1	0.388
**Not recorded**	14		5	19.2	19	7.36

	**Median (IQR)**	**Median (IQR)**
**ECOG performance status**	1 (1)	1 (1)
**Age at surgery (years)**	58 (14)	61 (9.5)

**Procedure**	**n**	**%**	**Procedure**	**n**	**%**
2-stage oesophagectomy	117		Whipple procedure	14	53.8
Total gastrectomy	48		Pylorus-preserving pancreaticoduodenectomy	7	26.9
Subtotal gastrectomy	23		Total pancreatectomy	2	7.69
Extended total gastrectomy	16		Central hepatectomy	2	7.69
LTA oesophagogastrectomy	22		Extended left hepatectomy	1	3.85
3-stage oesophagectomy	4		TOTAL	26	
Completion gastrectomy	1				
Distal sleeve gastrectomy	1				
**TOTAL**	232				

**Clavien-Dindo**	**n**	**%**	**n**	**%**	n	%
None	147		21	80.8	168	65.1
I	3		0	0	3	3.33
II	48		4	80	52	57.8
IIIA	8		0	0	8	8.89

IIIB	4		1	20	5	5.56
IVA	16		0	0	16	17.8
IVB	3		0	0	3	3.33
V	3		0	0	3	3.33
Total complications	85		5	19.2	90	

	**Median (IQR)**	**Median (IQR)**	**Median (IQR)**
**CCU LoS (days)**	4 (4)	2 (1)	3 (4)
**Total IP LoS (days)**	13 (5)	9.5 (8.75)	13 (5)

	n	%	n	%	n	%
**30-day mortality**	3		0	0	3	1.16
**90-day mortality**	5		0	0	5	1.94

**R0**	204		20	76.9	224	86.8
**R1**	28		6	23.1	34	13.2
**Not specified**	0		0	0	0	0

OG; Oesophago-gastric, HPB; Hepato-pancreato-biliary, LTA; Left Thoraco-abdominal, CCU; Critical Care Unit, IP; Inpatient, LoS; Length of Stay, R0; resection margins clear of microscopic evidence of carcinoma, R1; resection margins positive for microscopic evidence of cancer.

Table S4. Outcomes of low volume dyads operating on low risk cases.

	**OG**		**HPB**		**COMBINED**	
	**n**	%	**n**	%	n	%
**Male**	78	80.4	16	51.6	94	73.4
**Female**	19	19.6	15	48.4	34	26.6
**TOTAL**	97		31		128	

**ASA**	n	%	**n**	%	n	%
**1**	0	0	0	0	0	0
**2**	58	59.8	19	61.3	77	60.2
**3**	33	34.0	10	32.3	43	33.6
**4**	1	1.03	0	0	1	0.78<br>1
**Not recorded**	5	5.15	2	6.45	7	5.47

	**Median (IQR)**	**Median (IQR)**
**ECOG performance status**	1 (1)	1 (1)
**Age at surgery (years)**	57.5 (13.4)	58 (11.5)

**Procedure**	**n**	%	**Procedure**	**n**	%
2-stage oesophagectomy	43	44.3	Whipple procedure	17	54.8
Total gastrectomy	20	20.6	Pylorus-preserving pancreaticoduodenectomy	10	32.3

Subtotal gastrectomy	13	13.4	Mini-mesohepatectomy	1	3.23
LTA oesophagogastrectomy	11	11.3	Major Liver Resection	1	3.23
Extended total gastrectomy	4	4.12	Total pancreatectomy	1	3.23
3-stage oesophagectomy	2	2.06	Distal pancreatectomy and splenectomy	1	3.23
Transhiatal oesophagectomy	1	1.03	**TOTAL**	31	
Resection of O-J anastomotic recurrence	1	1.03			
Partial (fundal) gastrectomy	1	1.03			
Total splenectomy and distal pancreatectomy for single-site recurrence	1	1.03			
**TOTAL**	97				

**Clavien-Dindo**	**n**	%	**n**	%	**n**	%
None	65	67.0	21	67.7	86	67.2
I	1	1.03	0	0	1	2.38
II	14	14.4	9	90	23	54.8
IIIA	5	5.15	0	0	5	11.9
IIIB	2	2.06	1	10	3	7.14
IVA	7	7.22	0	0	7	16.7
IVB	3	3.09	0	0	3	7.14
V	0	0	0	0	0	0
Total complications	32	33.0	10	32.3	42	32.8

	**Median (IQR)**	**Median (IQR)**	**Median (IQR)**
**CCU LoS (days)**	3 (3)	2 (1.25)	3 (3)
**Total IP LoS (days)**	12 (6)	12 (7.75)	12 (6.25)

	**n**	%	**n**	%	**n**	%
**30-day mortality**	0	0	0	0	0	0
**90-day mortality**	1	1.03	0	0	1	0.781

	**n**	%	**n**	%	n	%
**R0**	87	89.7	23	74.2	110	85.9
**R1**	9	9.28	6	19.4	15	11.7
**Not specified**	1	1.03	2	6.45	3	2.34

OG; Oesophago-gastric, HPB; Hepato-pancreato-biliary, LTA; Left Thoraco-abdominal, CCU; Critical Care Unit, IP; Inpatient, LoS; Length of Stay, R0; resection margins clear of microscopic evidence of carcinoma, R1; resection margins positive for microscopic evidence of cancer.
